# chem^f^: A purely functional chemistry toolkit

**DOI:** 10.1186/1758-2946-4-38

**Published:** 2012-12-20

**Authors:** Stefan Höck, Rainer Riedl

**Affiliations:** 1 Institute of Chemistry and Biological Chemistry, ZHAW Zurich University of Applied Sciences, Einsiedlerstrasse 31, 8820 Wädenswil, Switzerland

**Keywords:** Functional Programming, *chem*^*f*^, Chemistry Toolkit, SMILES parser, Parallelization, Scala, Medicinal Chemistry

## Abstract

**Background:**

Although programming in a type-safe and referentially transparent style offers several advantages over working with mutable data structures and side effects, this style of programming has not seen much use in chemistry-related software. Since functional programming languages were designed with referential transparency in mind, these languages offer a lot of support when writing immutable data structures and side-effects free code. We therefore started implementing our own toolkit based on the above programming paradigms in a modern, versatile programming language.

**Results:**

We present our initial results with functional programming in chemistry by first describing an immutable data structure for molecular graphs together with a couple of simple algorithms to calculate basic molecular properties before writing a complete SMILES parser in accordance with the OpenSMILES specification. Along the way we show how to deal with input validation, error handling, bulk operations, and parallelization in a purely functional way. At the end we also analyze and improve our algorithms and data structures in terms of performance and compare it to existing toolkits both object-oriented and purely functional. All code was written in *Scala*, a modern multi-paradigm programming language with a strong support for functional programming and a highly sophisticated type system.

**Conclusions:**

We have successfully made the first important steps towards a purely functional chemistry toolkit. The data structures and algorithms presented in this article perform well while at the same time they can be safely used in parallelized applications, such as computer aided drug design experiments, without further adjustments. This stands in contrast to existing object-oriented toolkits where thread safety of data structures and algorithms is a deliberate design decision that can be hard to implement. Finally, the level of type-safety achieved by *Scala* highly increased the reliability of our code as well as the productivity of the programmers involved in this project.

## Background

Since there already exist a plethora of cheminformatics toolkits both open source and proprietary, one might wonder whether there is truly a need for another one. Most of the available toolkits such as the *Chemistry Development Kit*[1] or *OpenBabel*[2] are written in object-oriented languages using typical imperative concepts such as mutable data structures and opaque methods (see below) to implement chemical entities and algorithms. While the communities of many of the newer (and some of the older) programming languages start to appreciate the benefits of programming in a pure, referentially transparent fashion, these concepts have not yet had a big impact on software used in chemistry. We would therefore like to show some of the benefits of functional programming when applied to cheminformatics and talk about the type-safety and conciseness that can be achieved when using this programming paradigm.

### The dangers of side effects

Several widely used programming languages such as *Java*[3] or *C++* use object-oriented programing as a means to structure code and build reusable components. Such code is typically (but not necessarily) written in an imperative style, where statements represent actions the computer should carry out sequentially. Although it is regarded as a best practise to favor immutable data structures over mutable ones [4], imperative code is typically full of reassignment operations since these provide a convenient way to update fields in complex data structures. Mutable state can be hard to reason about when several seemingly unrelated parts of a program access and possibly modify the same piece of shared data. Special care needs to be taken when using mutable objects in parallelized algorithms [5].

Not only mutable state makes parallelization and reasoning about programs difficult: Accessing any kind of shared resources such as files or database connections can lead to unexpected behavior depending on a whole bunch of factors such as access rights, security settings, file locks and so on. Software components that access shared resources can be difficult to test and require special precautions when being accessed from several threads simultaneously. Although this has been known for quite some time, most programming languages make no distinction between methods that perform side effects by accessing shared resources or mutable state and functions that perform pure calculations just from their input parameters. Methods and objects written in these languages are therefore *a priori* unsafe to be used in parallel computations unless their documentation explicitly states differently. It would of course be much safer if one could rely on the compiler omitting errors or at least warnings when calling some unsafe method instead of having to trust in the accuracy of third party library documentation.

### Referential transparency [6]

An expression in a program is said to be *referentially transparent* when all it does is calculating its result from its input parameters (possibly by calling other referentially transparent functions) and *nothing else*. A referentially transparent function may not access or alter mutable state, nor may it perform any other form of side-effects. In an expression, a call to a referentially transparent function can always be replaced with the function’s result for the given set of parameters without altering the behavior of the program. A method that performs any number of side effects is said to be *opaque*. Referentially transparent functions offer several advantages over opaque ones: They are typically easier to reason about, can easily be tested and sometimes even proved to be correct, can be composed at will to create more complex functions (which are then still referentially transparent), and they can be safely used in parallelized algorithms. Since they need no access to resources other than their input parameters in order to perform their tasks, they make for highly reusable building blocks for creating more complex functionality.

Since mutability breaks referential transparency, referentially transparent expressions can only work with immutable data structures. This can make writing referentially transparent expressions cumbersome in typical imperative languages where mutability is the rule rather than the exception and the language’s syntax provides only marginal support for working with deeply nested immutable data structures.

### Functional programming

One programming paradigm that greatly facilitates writing referentially transparent functions and using immutable data structures is the one of *functional programming*. Functional programming languages were designed with referential transparency in mind, and they encourage a more declarative style of programming without the control statements and value assignments typically found in imperative ones. In functional programming, functions are first class values that can be passed as parameters to other functions (which are then called higher-order functions) or be the results of other functions. Functional programming languages typically are statically typed but use type inference to prevent type annotations from cluttering the code. Together with the above mentioned higher order functions this can lead to highly concise code. For instance, iterating over a list of integers to produce a list of their squares might look like this in an imperative language (the code is written in *Scala*[7], a multi-paradigm language that is described in more detail below):

def squares (is: List[Int]): List[Int] ={val squares = new ListBuffer[Int]

{for i <‐ is}{squares += i ⋆ i}

squares.toList}

It is typical for an imperative program to update a mutable data structure during iteration. The same program written in a more functional style might look like this:

def squares(is: List[Int]) =is map (x ⇒ x ⋆ x)

Here we make use of the higher-order function map that takes another function as its parameter, which is then applied to each element in the list. Not only is this expression much shorter than its imperative counterpart, function map also describes such a common pattern when working with collection-like data structures that it provides a plethora of functionality all of which can be used with almost no syntactic overhead.

Given that objects of type List are immutable, both code examples shown above are referentially transparent. Although the imperative example uses a (mutable) ListBuffer to accumulate its result, this is an implementation detail hidden behind the function’s interface.

Some functional programming languages like *Haskell*[8] enforce referential transparency through their type system and can therefore exploit its advantages to their full extent. This includes several optimization techniques during compilation as well as memoization of a function’s results for given parameter sets at runtime.

Although the advantages of functional programming have been known for a long time [9], many of the concepts described above where not or only partially implemented in the mainstream programming languages and a major part of the programming community does not exploit the benefits they provide.

### Functional programming and chemistry

We believe that the above mentioned aspects of functional programming make this paradigm very well suited for writing scientific applications where the main focus often lies on running complex calculations rather than performing long cascades of side effects. For instance, the only side effects involved when running a high throughput screening with a typical command line based docking software are reading from and writing to files (and possibly to the console). The rest is pure, stateless calculations that are well suited to be written in a purely functional manner and run in parallel on a multi-core system. When we set out to write the first prototype of our in-house data management tool *CyBy*^*2*^[10], we used *Java* as the language of choice, since there already existed several chemistry toolkits written in that language. It was only after *CyBy*^*2*^ was already running on our servers and several hard to find bugs took us hours if not days to get rid of that we decided to go for a change in the style of programming. We decided to give the *Scala* programming language a try since programs written in *Scala* run on the Java Virtual Machine (JVM) and existing *Java* libraries can be accessed directly from within *Scala* source code. *Scala* is a modern multi-paradigm programming language that is fully object-oriented with a strong support for typical concepts from functional programming such as higher-order functions, type inference, and pattern matching. It also has one of the most sophisticated and expressive type systems written so far [11]. *Scala* is not a pure functional programming language that enforces referential transparency through its type system (this can be achieved by using the effect system provided by *scalaz* though; see below), but programming in a pure manner as well as the use of immutable data structures are strongly encouraged and facilitated by the language.

While our style of programming moved from imperative to functional, we found it cumbersome and sometimes error prone to interact with the methods and classes coming from third-party *Java* libraries that make heavy use of mutable data structures and side effects. We therefore started looking for cheminformatics toolkits written in a more functional style and were surprised to find only one such toolkit at an early stage written in *Haskell*[12]. It was this lack of purely functional third-party libraries that made us decide to go ahead and write our own toolkit. In this article we present our first experiences along that road. We will show how molecular graphs can be represented using persistent, immutable data structures and how parsing input can be done in a referentially transparent way leading to highly reusable code that can be run in parallel out of the box. We do this by implementing a purely functional SMILES parser [13], a reasonable complex task to get a feeling for some of the higher-order abstractions typically used in functional programming. Along the way we will show how to deal with input validation, error handling, and parallelization in a purely functional, type-safe manner.

### A short introduction to *Scala*

In the remainder of this article we will often show snippets of code to discuss the aspects of functional programming when encountering certain problems. We will use *Scala* as the implementing language for the reasons given above. It will not be necessary to be fluent in *Scala* to grasp the concepts being presented here, yet we think that a short introduction to this deep language and its syntax will make it easier to understand the examples in this article. For a more thorough introduction please see [14]. A note on terminology: In object-oriented programming languages, subroutines are typically called *methods* without making a distinction between those that perform side effects and those that do not. For the remainder of this article we use the term *method* for side-effect performing subroutines and *function* for pure calculations.

#### Basic syntax

*Scala*’s syntax is similar to the one of *Java* and other *C*-like languages. Statements are usually enclosed in curly braces and delimited by semicolons. Many of these structural delimiters can be inferred by the compiler leading to less cluttered code when writing the short expressions typically found in functional programming. The following two function definitions both compile:

def square (i: Int): Int = {i ⋆ i;}

def square (i: Int) = i ⋆ i

As can be seen in the example above, type annotations come after variable names and can in many cases be inferred by the compiler.

In terms of object-oriented programming, *Scala* – like *Java* – has classes and interfaces (which are called *trait*s and can contain method implementations and state). *Scala* also has singleton objects, which are defined using the object keyword. There are no static methods in *Scala*: Every method definition belongs to a class or an object. There is the special case of *companion objects*, which have the same name as their companion class and are typically used to define class-related constants and ’static’ functions:

class AClass {…}

object AClass {//a factory methoddef create: AClass = …}

A special case that is often used in functional programming to define algebraic data types [15] is the sealed keyword: Subclasses of a sealed class or trait must be defined in the same file as the sealed class itself.

#### Function literals

In *Scala*, functions are first-class values and the language provides a lot of syntactic sugar to help declaring them. A typical function literal looks as follows:

val add: (Int,Int) ⇒ Int =(a: Int, b: Int) ⇒ a + b

(Int,Int) ⇒ Int is a type annotation: A function from two integers to another integer. Type inference lets us remove some code duplication:

val add: (Int,Int) ⇒ Int =(a,b) ⇒ a + b

If both parameters appear in the function’s body only once and in the same order as in the function’s parameter list, we can replace them with placeholders:

val add: (Int,Int) ⇒ Int = _ + _

We can use function literals to create anonymous functions as parameters for higher-order functions. For instance, we can double all the integers in a list like so:

def dbl (xs: List[Int]) = xs map (2⋆)

#### Lazy evaluation and by-name parameters

By default, *Scala* is a strict language, meaning function parameters are evaluated before the function’s body is executed. However, *Scala* facilitates lazy evaluation of values as well as by-name function parameters:

lazy val aVal = …

def aMethod (byNameParam: ⇒ Int) …

Values marked with the *lazy* keyword are not evaluated until they are accessed for the first time. They are evaluated only once. By-name parameters are also evaluated only when needed, but unlike lazy values they are reevaluated each time the parameter is accessed.

#### Parametric polymorphism

Similar to *Java*, *Scala* supports subtype polymorphism as well as parametric polymorphism (aka *generics*). Type parameters are declared within brackets and can be annotated with variance annotations:

trait Invariant[A]

trait Covariant[+A]

trait Contravariant[‐A]

Variance describes subtyping for classes with type parameters. In the case of covariance, Covariant[A] is a subtype of Covariant[B] if A is a subtype of B. On the other hand, Contravariant[A] is a subtype of Contravariant[B] if A is a supertype of B.

In contrast to many other programming languages, *Scala* does not stop at first-order parametric polymorphism, but also allows type constructors as type parameters (higher-kinded types) [16]. Only these constructs make the implementation of some of the more powerful type classes possible (see below).

#### Case classes and pattern matching

In the tradition of other functional programming languages, *Scala* supports *pattern matching*, which can be used to query and match even complex nested data structures. *Case Classes* are like other classes in *Scala* but are enhanced automatically by pattern matching functionality by the compiler. Also, constructor parameters are automatically added as immutable fields with public scope to case classes. For instance, a simple setup of chemistry-relevant classes may look as follows:

sealed abstract class Element (val atomicNr: Int)…

case object C extends Element(6)case object N extends Element(7)…

case class Isotope (element: Element,massNumber: Int)

case class Atom (isotope: Isotope,charge: Int)

Pattern matching can now be used to count all carbon atoms in a molecule:

def isCarbon (a: Atom) = a match {case Atom(Isotope(C, _), _) ⇒ truecase _ ⇒ false}def carbonCount (m: Molecule): Int =m.atoms count isCarbon

Underscores act as placeholders that match any value while lower case identifiers can be used to access a value in the following code block:

def carbonMass (a: Atom): List[Int] =a match {case Atom(Isotope(C, mass) _) ⇒List(mass)case _ ⇒ Nil}

The first case statement matches carbon atoms of an arbitrary mass number and the mass number is returned wrapped in a List. In case of non-carbon atoms, the function returns the empty list Nil.

#### Implicits and the *pimp my library*-pattern

*Scala* supports implicit conversions from one type to another in order to enrich classes from third-party libraries with additional functionality. This is called the *pimp my library* pattern [17] within the *Scala* community.

For instance, if one wanted to add the ability to calculate the exact mass distribution to a class Molecule from a third-party library that does not yet provide such a function, one would first have to define a wrapper class that defines the function in question together with an implicit conversion from Molecule to MoleculeWrapper:

class MoleculeWrapper (m: Molecule) {def exactMasses = …}

implicit def mol2Wrapper (m: Molecule) =new MoleculeWrapper(m)

It is now possible to call exactMasses on Molecules as if the function was provided by class Molecule itself:

def printMasses (m: Molecule) {m.exactMasses foreach println}

#### Type classes

In addition to implicit conversions, *Scala* also supports implicit parameter lists for functions. These parameters need not be provided explicitly if an implementation of the type in question can be found in implicit scope. One use case for implicit parameters is the definition of *type classes*[18] similar to those found in *Haskell*. Type classes are a way to implement ad-hoc polymorphism (method overloading) in purely functional programming languages. For instance, the *scalaz* library (see below) defines a type class Equal for type safe structural equality checking as follows:

trait Equal[‐A] {def equal(a1: A, a2: A): Boolean}

Via the *pimp my library*-pattern, *scalaz* then adds operators $\overset{?}{=}$ and to all types. These are defined in wrapper class Identity and are implemented as follows:

def$\overset{?}{=}$(a: A)(implicit e: Equal[A]): Boolean = e equal (value, a)def and$\neq \overset{˘}{\text{a}}$(a: A)(implicit e: Equal[A]) : Boolean = !($\overset{?}{=}$(a))

All objects for which an instance of type class Equal can be found in implicit scope can now be compared in a type-safe manner using these operators. There exists a shorthand notation using *context bounds* for functions that require an instance of a type class with a single type parameter to be in implicit scope:

def isEqual[A:Equal] (a1: A, a2: A) = a1 $\overset{?}{=}$a2

#### The *scalaz* library [19]

Several of the more advanced techniques and abstractions found in functional programming did not make it into the *Scala* standard library. Many of these are provided by *scalaz*, a collection of type classes and purely functional data structures. Many of the type classes defined in *scalaz* come from the world of *Haskell* where a lot of information on things like Monoids, Functors, Applicatives, Monads and others are available. For a gentle and highly entertaining introduction to some of these concepts see [20].

## Results and discussion

We studied the aspects and benefits of functional programming in cheminformatics by first designing an immutable data structure for representing molecules and then implementing a SMILES parser in accordance with the OpenSMILES specification [21]. We then tested how immutable data structures and referentially transparent functions can be used in parallelized bulk operations out of the box and had a closer look at our algorithms in terms of performance. The full source code of the data structures and algorithms discussed in this section is available in Additional file 1. We also created a repository on *github* to further advance the development of our toolkit. The repository can be found at https://github.com/stefan-hoeck/chemf.

### Molecules as immutable data structures

There exist many ways to represent a chemical molecule in a computer, many of which depend on the concrete aspects one is focussing on. *Lewis* structures of molecules are typically represented as undirected labeled graphs with the atoms at the vertices and the bonds at the edges [22].

#### Immutable undirected graphs

We used a simple unlabeled connectivity list as the basis for our molecular graphs. The graph’s vertices are numbered from 0 to order ‐ 1 where order is the total number of vertices.

sealed trait Graph {def order: Intdef edges: Set[Edge]}

We provided a couple of helper functions for adding and removing edges and vertices, all of which return a new, immutable copy of the updated graph. Trait graph is backed by an immutable Set of Edges, which provides effective constant time add, remove, and lookup operations [14]. Since all edges in the graph potentially have to be adjusted when removing a vertex, this operation takes linear time with respect to the size (= number of edges) of the graph.

Some of the typical graph algorithms make heavy use of adjacency lists [23] which we provided as a lazily initialized value in trait Graph. Adjacency lists were implemented using *scalaz*’s ImmutableArray providing fast constant time random access.

lazy val adjacencyList: ImmutableArray[Set[Int]] = …

Edges in graphs were implemented as ordered pairs of integers:

sealed trait Edge {def a: Intdef b: Int}

To make it easier to compare and sort edges, we added an invariant to the edge class stating that vertex b is the higher of the edge’s two vertices. Edges can therefore only be instantiated via a factory method to guarantee that this invariant is fulfilled. This very basic graph implementation has a low memory footprint and can be used to determine structural parameters such as atom and bond count as well as number and size of rings in a molecule.

#### Molecules as labeled graphs

In order to represent the *Lewis* structures of molecules, we needed to label the vertices and edges of the graph with additional information. We therefore introduced trait LGraph:

trait LGraph[+E,+V] {def graph: Graphdef vLabel (v: Int): Vdef eLabel (e: Edge): E}

Trait LGraph is parameterized over the types of both the edge and vertex labels, giving us maximum flexibility when working with different representations of molecules. The trait again provides functions for adding and removing edges and vertices as well as some higher order functions like map and foldLeft typically found in functional data structures. We also implemented type classes Functor, Foldable, and Traverse (all defined in the *scalaz* library) for LGraph, giving us a lot of functional power when iterating over the elements of LGraph as we will show further below.

The core functionality of LGraph was implemented using a private case class LgImpl:

private case class LgImpl[+E,+V] (graph: Graph,vertices: IndexedSeq[V],eMap: Map[Edge,E]) extends LGraph[E,V] {def vLabel (v: Int) = vertices (v)def eLabel (e: Edge) = eMap (e)}

Implementing functions graph and eLabel was straight forward. For function vLabel there were several possible implementations to consider. We decided to use an IndexedSeq to store vertex labels, which provides effective constant time append, prepend, and random access operations. Using an Array would give us (fast) constant time random access but other operations might be considerably slower. Later it might be convenient to provide two or more different implementations depending on the performance characteristics needed by the algorithms working with our graphs.

#### Atoms and bonds

We favored composition over inheritance when implementing the core classes needed to represent typical chemical entities. At the root stands class Element:

sealed abstract class Element (val atomicNr: Int)

object Element {case object H extends Element (1)case object He extends Element (2)…}

A lot of additional information can be requested from Element objects, all of which is lazily loaded from the *Blue Obelisk Element Repository*[24].

Next come the isotopes, which unlike the elements were not implemented as constants to give us a bit more flexibility when working with newly-discovered or purely hypothetical isotopes:

sealed trait Isotope {def element: Elementdef massNumber: Option[Int]}

Objects of type Isotope either represent a natural mixture of the isotopes of a given element or a single isotope with a concrete mass number. Value massNumber may therefore be undefined, which is represented by its type: Option[Int]. Option[A] is an algebraic data type with two possible values: Some(a) with a being a wrapped value of type A, or None. It is used as a type-safe alternative to using null as the result of unsuccessful calculations or for undefined values. Using Option makes checking for null and throwing NullPointerExceptions in client code obsolete.

Again additional information about isotopes is lazily loaded from a file, this time from the *Blue Obelisk Isotope Repository*. Since in chemistry we are usually dealing with the same (small) set of isotopes most of the time and since our isotopes are immutable they make good candidates for applying the *Flyweight* pattern [25]. Instead of creating many new objects when assembling a molecular graph, the most commonly used isotopes are stored in an array and returned when new isotopes are requested by clients. Therefore we made isotopes only available through two factory methods:

object Isotope {def apply (e: Element): Isotope = …

def apply (e: Element, mn: Int): Isotope = …}

*Scala* provides a shorthand notation for calling functions named apply. For instance a carbon isotope can be requested like so:

val c = Isotope(Element.C)

Here we truly profited form using immutable objects: Using the *Flyweight* pattern keeps the memory footprint of our molecules low while letting us store a lot of additional information for all isotopes and elements that has to be loaded and calculated only once.

We were now able to define a basic Atom implementation:

case class Atom (isotope: Isotope,charge: Int,hydrogens: Int,stereo: Stereo)

Further fields like hybridization or coordinates might be necessary for some algorithms but instead of cluttering Atoms with lots of optional fields, we argue that in those cases a more specialized class should be used which will lead to an increase in terms of type-safety. An algorithm requiring coordinates for atoms might be able to work with an object of type LGraph[Bond,Atom3D] but will reject LGraphs with other vertex types.

We distinguished between the different bonds possible in *Lewis*-structures via a couple of constants:

sealed trait Bondobject Bond {case object Single extends Bondcase object Double extends Bond…}

Molecules can now be defined as a simple type alias:

type Molecule = LGraph[Bond,Atom]

#### Some basic operations on molecules

We implemented a couple of basic operations on molecules to demonstrate some of the higher order concepts typically found in functional programming.

First, we wanted to calculate the molar mass of a chemical compound. For that we defined function mass that calculates the mass of a single Atom together with the masses of its implicit hydrogen atoms:

def mass (a: Atom): Double =a.isotope.mass + a.hydrogens ⋆ H.mass

The molar weight of a molecule could then be calculated as follows:

def molarMass (m: Molecule): Double =m foldMap mass

Method foldMap comes from the *scalaz* library and is similar to the reduce function described by *McBride* and *Paterson*[26]. It requires two type class implementations to be in implicit scope: Foldable for the data structure over which we iterate (in our case this is LGraph) and type class Monoid for the return type of the function we pass as the sole parameter to foldMap. Foldable defines several functions for accumulating values when iterating over a data structure. We will not discuss it in more detail here but refer the reader to the *scalaz* source code. Type class Monoid has many applications in programming. It defines two functions: Function append defines some form of combining two values into a single value of the same type, while zero is the neutral element of this operation such that zero append x == x and x append zero == x. Here is the Monoid implementation for floating-point addition:

implicit val DoubleMonoid =new Monoid[Double] {val zero: Double = 0.0Ddef append (a: Double,b: ⇒ Double) = a + b}

While it is nice to implement molarMass with just a single line of code our implementation is not very safe. It could be that mass is not known for all possible isotopes. This should be reflected in the function’s return type, and the function was adjusted accordingly:

def mass (a: Atom): Option[Double] =

We dealt with the possibility of failure by returning a value of type Option[Double]. Interestingly, *scalaz* defines a Monoid instance for Options of Monoids. We therefore only had to change the return type of molarMass and our code still compiled and behaved as expected:

def molarMass (m: Molecule): Option[Double] = m foldMap mass

The molar mass of a molecule is now wrapped in a Some if the masses of all its isotopes were known, otherwise the function returns None. Unlike the many methods written in *Java* that return null in case of an unsuccessful calculation or an undefined value, function molarMass is absolutely type-safe and we can rely on the compiler to make sure we deal with the possibility of an unknown molar mass properly.

We could have gone even further and use *scalaz*’s Validation class instead of Option. In that case we would have either gotten the molar mass wrapped in a Success or a list of error messages explaining exactly for which isotopes the mass could not be determined. Even in this case we would have been able to use foldMap to accumulate the validated isotopic masses. We used Validation when implementing our SMILES parser and will describe its applications further below.

Next, we implemented a function to determine the total formula of a molecule. We represented this as a Map from Isotope to Int. First, we generated such a Map for a single atom:

type Formula = Map[Isotope,Int]

def atomFormula (a: Atom): Formula =Map(a.isotope ⇒ 1) ++ ((a.hydrogens > 0) ?Map(Isotope(H) ⇒ a.hydrogens) |Map.empty)

Since *scalaz* defines a Monoid for Maps for whose value type a Monoid is defined, we again were able to use foldMap:

def formula (m: Molecule): Formula == m foldMap atomFormula

We wanted to show with these examples how common abstractions like Monoid can be used to write highly reusable functions like foldMap. We also argue that it is almost impossible to achieve the same levels of abstraction and code reuse by using object-oriented concepts like inheritance instead of type classes.

### A purely functional SMILES parser

Now that we had a way to handle molecular graphs it was time to implement some parsing capabilities for text-based user input. SMILES is well suited for this task since its syntax is easy to learn and SMILES strings for reasonably small molecules can be generated by hand without the help of a computer.

In the following sections we will describe several issues that might come up when reading user input. Our focus always was more on type-safety, referential transparency, and code reuse than on optimizing performance, which we believe can still be done once one has a properly running application.

#### First things first: The parser as a finite state automaton

Parsing a SMILES string means interpreting a single line of data one character at a time while accumulating an increasingly complex molecular structure. We therefore needed two things: A data type representing the accumulated molecule and a finite state automaton (FSA) where each state of the automaton takes a character and the last molecule as input and returns an updated molecule together with a new automaton state. The following code listing shows how we modeled such an FSA:

type FARes[A] = (FAState[A], A)sealed trait FAState[A] {def next(a: A, c: Char): FARes[A]}

object FAState {def apply[A](f: (A,Char) ⇒ FARes[A]): FAState[A] = new FAState[A] {

def next(a: A, c: Char) = f(a, c)

}}

Trait FAState represents the finite automaton’s actual state. Since this class describes a very general concept and might be used for different types of parsers, we abstracted over the type of the accumulated data using a type parameter. The return type of function next is a pair consisting of a new FAState and the updated data object. We defined a general-purpose factory method apply that takes a single function as its argument in the companion object of FAState.

We then defined function parse, which takes a single string and an initial automaton state and returns the parsed value:

val EOT = '0̆004'

@scala.annotation.tailrecdef parse[A] (s: String, fas: FAState[A], a: A): A = s match {case "" ⇒ fas.next(a, EOT)._2case cs ⇒ fas.next(a, cs.head) match {case (newFas, newA) ⇒parse(cs.tail, newFas, newA)}}

We used a tail-recursive function for parsing strings. A function is tail-recursive if the only place the function calls itself is the last operation of the function [14]. The *Scala* compiler can optimize tail-recursive functions, so that in our parser the call stack will not overflow even when parsing strings with thousands of characters. Annotation tailrec is a safety measure to make sure that our function is indeed tail-recursive. If this is not the case, the code will not compile. The parser is informed that the end of the string is reached by passing value EOT (end of transmission) as a parameter. This is necessary since the parser might still expect further input. For instance, if the last character in a SMILES string is 'C' the parser will only know whether a carbon or chlorine atom must be added to the molecule upon parsing the next character in the string. If this happens to be EOT, a final carbon atom should be added.

The classes and functions presented here describe a general purpose incremental string parser. So far they are completely unrelated to molecules and chemistry.

#### Enhancing the *Builder* pattern with type classes

We distributed the responsibility for parsing SMILES strings over three classes. First, we defined type class SmilesBuilder[A] whose instances know how to accumulate molecular information using objects of type A. A SmilesBuilder provides all actions required when parsing a SMILES string:

trait SmilesBuilder[A] {type STrans = A ⇒ Aval empty: Adef addAtom (a: SmilesAtom): STransdef clear: STransdef closeBranch: STransdef openBranch: STransdef ring (i: Int): STransdef setBond (b: Bond): STransdef setDbStereo (c: Char): STrans}

It is important to note that the builder was designed to work on immutable data structures. All its methods return functions, while in an imperative implementation using mutable state their return type would be Unit (similar to void in *Java*).

Next, class SmilesParser[A] is responsible for traversing the SMILES string and interpreting the characters it encounters. Instead of directly accumulating a molecular graph, it delegates data accumulation to an implementation of SmilesBuilder[A], which must be in implicit scope when creating a new SmilesParser. Parts of the implementation of SmilesParser are shown below: 

First, we defined type alias STrans, which is just a function from one accumulated molecule to the next. The main entry point for parsing a SMILES string is the initial automaton state char, which performs a pattern match against the character to be parsed. Seven cases had to be distinguished: EOT returns the accumulated molecule without further modifications together with a dummy parser, 'C' and 'B' await the next character in order to decide between adding one of two possible atoms, '[' will start bracket accumulation while the next two cases have to do with ring formation. The final catch-all pattern looks up the character in map unique and applies the returned state transformer to molecule a. Map unique contains all characters that can be encountered outside of brackets in a valid SMILES string other than the few special ones just described.

For interpreting the detailed data coming in brackets, we used regular expressions together with *Scala*’s pattern matching capabilities to great effect. Thus, the whole parser consisted of less than one hundred lines of code and should be able to parse all strings in accordance with the *OpenSMILES* specification [21] with the exception of chemical reactions, where the participating molecules are delimited by character '>'. Since we believe that a reaction is not the same thing as a molecule, parsing reactions will have to be treated separately. This is trivial though, since we can just split the string into three (possibly empty) parts at the proper places, and treat each part as a separate molecule. Finally we needed a class to represent the accumulated SMILES state:

type AtomInfo = (Int, Boolean)type RingInfo = (AtomInfo, Option[Bond])type Rings = Map[Int,RingInfo]

case class SmilesMol (graph: LGraph[Bond,SmilesAtom],keep: Boolean,stack: List[AtomInfo],bond: Option[Bond],dbStereo: Option[Char],rings: Rings)

Field graph stores the growing molecular graph. Flag keep is set to true when a new side chain is started, in which case the next atom’s index together with is information about aromaticity will be added to the atom stack. Fields bond and dbStereo are used when explicit information about bonds is encountered in a SMILES string, and map rings represents all currently opened rings including the type of the ring bond (if specified) and whether the first atom at the ring bond is aromatic. We thought that these fields will hardly ever be used outside of class SmilesMol, therefore we did not define a new data type for each of them but used tuples and type aliases instead. For class SmilesMol an implementation of SmilesBuilder was written in about one hundred lines of code so that we were finally able to define our first concrete SMILES parser:

val Default = SmilesParser[SmilesMol]

What we applied here is almost exactly the *Builder* pattern described by the famous *Gang of Four*[25]. Class SmilesParser plays the role of the *director* with type class SmilesBuilder[A] being the *builder* for product type A. The only difference is that we used *Scala*’s type class mechanism to bring the *builder* to the *director*. The big advantage of the *builder* pattern is the decoupling of parsing and state accumulation. When parsing a SMILES string we might not always be interested in accumulating a full molecular graph. In that case we might go for a completely different product type A. All we had to do in such a case would be writing a new implementation of SmilesBuilder[A] which is typically much easier than writing a new parser from scratch.

#### Implicit hydrogen detection

A SMILES string has not only to be parsed according to syntactic rules, it usually also needs to be interpreted chemically. Typical additional calculations include perceiving aromaticity, interpreting stereochemical information, and detecting implicit hydrogen atoms. Implementing the latter was pretty straight forward: For the organic subset atoms, SMILES defines one or more valid default valences. We therefore only had to count the total number of bonds to organic subset elements and add implicit hydrogen atoms until we reached the next higher valid valence:

def implicitHydrogens (bs: List[Bond], e: Element): Int = {def calc (v: Int): Option[Int] =valences get e flatMap (_ find (v<=))

calc (bs foldMap (_.valence)) get}

private val valences: Map[Element,Seq[Int]] = Map(B ⇒ Seq(3),C ⇒ Seq(4),N ⇒ Seq(3,5),…)

We used foldMap to sum up the valences of all bonds connected to the atom in question (this list is directly available from the molecular graph) and then search for the lowest valid valence equal to or greater than this value. The difference between the two are the implicit hydrogen atoms.

While the above code is nice and short, it goes horribly wrong in the presence of aromatic bonds. There is no single valence defined for an aromatic bond therefore they need to be interpreted on a case by case basis. We enhanced the above algorithm to check for the presence of aromatic bonds by first sorting list bs (aromatic bonds were at the head of the list after sorting) and then performing several pattern matches taking up about fifteen lines of code in total. This allowed us to finally transform a graph of type LGraph[Bond,SmilesAtom] to one of LGraph[Bond,Atom] which could then be used to calculate the molar mass and total formula of the parsed molecule using the functions described earlier in this article.

Using function implicitHydrogens it was straight forward to transform a SmilesMol to a Molecule and we defined function toMolecule for just that purpose:

object SmilesMol {def toMolecule (sm: SmilesMol): Molecue = …}

### Functionally handling exceptions: The Validation Applicative Functor

We have stressed the usefulness and safety of using referentially transparent functions several times in this article. At the same time, our SMILES parser is not referentially transparent at all. For instance, the following expression in our parser will throw an exception if character c is not found in map unique:

lazy val char: FAS = FAState[A]((a, c) ⇒ c match {…case x ⇒ (char, unique(x)(a))})

This is not a type-safe solution. Clients of our code would have to rely on the documentation of our library in order to know what kinds of exceptions to expect when using our parser. We will now describe an alternative strategy for dealing with exceptions that is type-safe, purely functional and composable at the same time.

We have seen how the Option data type can be used in cases where a calculation might have no valid result. While this is already an improvement in terms of type-safety, it has one drawback: We cannot tell the client of our function what exactly went wrong. What we would like to have is a data type that lets us return the results of successful calculations while returning some information about what went wrong in case of a failure. We have actually two options to get this behavior: The *Scala* standard library defines the algebraic data type Either[A,B] which has two subclasses Left[A,B] and Right[A,B] where a Left wraps a single value of type A and a Right one of type B. By convention a Left is returned in case of a failed calculation and a Right in case of a success. While this is already very useful, Either was not designed to accumulate error messages. If we parsed a file of many strings using function traverse (see below) with Either[String,SmilesMol] as the return type, our program would stop at the first invalid string encountered in the file returning a single error message wrapped in a Left. The clients of our parser would not know if there were other erroneous SMILES strings in the file until the one that stopped the parser was fixed and the application run again. If possible, clients typically want to know about all things that went wrong during a calculation. This is especially the case when running bulks of independent calculations.

An alternative to Either is provided by *scalaz* in form of the Validation[E,A] data type. Again, two subclasses are available: Failure[E,A] which wraps a single value of type E and Success[E,A] wrapping a value of type A.

Unlike Either, Validation was designed to accumulate failures. We will show how this is done in a moment. First we had to change parts of our parser so that they no longer threw exceptions but returned validated values of type Validation[NonEmptyList[String],A] instead. A Non‐ EmptyList is just that: A list that contains at least one value. It is provided by *scalaz* and is often used as a means to accumulate error messages.

#### A referentially transparent SMILES parser

First, we had to redefine some of our type aliases:

type ValRes[+A] =Validation[NonEmptyList[String],A]

type FARes[A] = ValRes[(FAState[A], A)]

trait SmilesBuilder[A] {type STrans = A ⇒ ValRes[A]…}

sealed abstract class SmilesParser[A] {type STrans = A ⇒ ValRes[A]…}

Of course we had to adjust some of the functions in classes SmilesBuilder and SmilesParser. For instance, the catch all case in FAStatechar was changed as shown below: 

unique get c returns an Option[STrans]. The two possible cases Some and None are handled in function fold: If a state transformer was found, it is applied to the last state a and the validated result is mapped to a pair consisting of the next automaton state and the updated molecule. This pattern came up several times in our SMILES parser therefore we moved it to helper function next. If no state transformer was found, the parser returns an error message wrapped in a Validation. Other functions in SmilesBuilder and SmilesParser were adjusted accordingly.

Finally, we adjust function parse in the companion object of class FAState as shown in the following code listing:

This time we did the tail recursion using an inner function, since we also wanted to keep track of the position in the SMILES string. That way, if a string cannot be parsed, all error messages are adjusted to include the exact position at which the parsing failed.

We also adjusted function toMolecule in the companion object of class SmilesMol to return validated molecules, since some errors in a SMILES string can only be detected upon implicit hydrogen calculation. We were then able to go directly from a string to a validated molecule by defining function smiles:

def smiles (s: String): ValRes[Molecule] =SmilesParser.Default(s) flatMapSmilesMol.toMolecule

Function flatMap is a more powerful version of function map (which can actually be expressed in terms of flatMap) in that it does not take a function from A to B as its parameter but one from A to ValRes[B]. By the way, when using flatMap the error messages of the two functions will not be accumulated since the second can only be called if the first was successful.

In order to provide some human-readable output for our molecular graphs, we implemented type class Show for trait LGraph. We could then test our parser directly from the console by using the following function:

def smilesShow (s: String): String =smiles(s) fold (_.list mkString " n",_.shows)

This is typical when working with types like Option and Validation: At one point one has to break out of the context and either handle the successfully calculated results or deal with the accumulated error messages. In this case we do just that by converting both possible outcomes into strings: In case of a failed calculation we collect all error messages in a single string (one error per line), in case of a success we present the molecular graph in human readable form. Both possible outcomes are shown in Tables 1 and 2 for several input strings.

**Table 1:** Output when parsing valid SMILES strings

**CCO**	**[cH+]1cc1**	**[NH3+][C@H](C)C(=O)[O-]**
LGraph:	LGraph:	LGraph:
0: CH3	0: CH(+1)	0: NH3(+1)
1: CH2	1: CH	1: CH(@)
2: OH	2: CH	2: CH3
		3: C
0 - 1: -	0 - 1: :	4: O
1 - 2: -	0 - 2: :	5: O(-1)
	1 - 2: :	
		0 - 1: -
		1 - 2: -
		1 - 3: -
		3 - 4: =
		3 - 5: -

Molecular graphs are displayed as lists of atoms followed by lists of bonds. Each bond shows the indices of the connected atoms followed by the SMILES symbol representing the type of the bond.

**Table 2:** Output when parsing invalid SMILES strings

**Input**	**Message**
CCuCC	Pos. 3 in CCuCC: Unknown character in SMILES-String: u
C%12CCCC%1	Pos. 11 in C%12CCCC%1: % is not followed by two digits
C#OC	Invalid bond set for element O: Triple,Single

In the last example no position is given since this failure did not happen during parsing but during implicit hydrogen detection which is a separate algorithm and therefore has not notion of a ’position in a string’.

#### Bulk operations

This section describes some of the more advanced techniques used in functional programming. These were described in greater detail by *McBride* and *Paterson*[26] as well as *Gibbons* and *Oliveira*[27].

Given a file containing a large number of molecules each as a SMILES string on a separate line, we were now able to parse the whole sequence of lines in a single line of code:

import SmilesParser.Default

def bulkParse (ls: Seq[String]) =ls traverse Default.parse

The result of bulkParse will either be a sequence of SmilesMols wrapped in a Success or a NonEmptyList of error messages wrapped in a Failure. Method traverse is again provided by *scalaz* and has the following quite daunting signature:

def traverse[F[_],B](f: A => F[B])(implicit a: Applicative[F],t: Traverse[M]): F[M[B]] = …

It is added via the *pimp my library* pattern to an object of type M[A] and takes a function from A to a value of type B in a context F as its argument. It also takes two implicit parameters: An instance of type class Traverse must be defined for type constructor M (which is usually some kind of container such as a list or a tree) and one of type class Applicative for context F. Method traverse is highly versatile in that it not only abstracts over the input and output types of a function but also over the container over which we iterate as well as the context F in which the calculation runs. We will not describe how these type classes are defined and implemented any further but refer the interested reader to the articles given at the beginning of this section as well as the *scalaz* source code. Suffice to say that the Applicative for Validation requires a Semigroup (which like a Monoid defines a function append but without the zero value) to be defined for its error type E. Errors are then accumulated via Semigroup append. For instance there is a Semigroup for NonEmptyLists (append simply being list concatenation).

One good thing about Applicatives is that they compose very nicely. It can be shown that both the product (M[_],N[_]) and the composition M[N[_]] of two Applicatives M[_] and N[_] are again Applicatives [26]. This gives us incredible flexibility in our code. We will demonstrate this by adding another piece of information to our error messages: The line number at which a certain error occurred. To keep track of the actual line number, we use the State Monad [28]. We define a couple of new type aliases together with function parseLine:

type IntState[A] = State[Int,A]type ValIntState[A] = IntState[ValRes[A]]

implicit val ValIntStateApplicative =Comp.CompApplicative[IntState,ValRes]

def parseLine[A] (f: String ⇒ ValRes[A]): String ⇒ ValIntState[A] =s ⇒ state(i ⇒ (i + 1, f(s) fold (_ map ("Line %d: %s" format (i, _)) fail,_.success)))

Method parseLine parses a string and if this fails, prepends the actual line number to all error messages. At the same time the line number is increased by one. Since we are working with a composition of Applicatives, the *Scala* compiler needs some help to figure out that ValIntState is still an Applicative. *scalaz* defines function CompApplicative for just this purpose. We can now define a function parseSmilesLine and use it again in a bulk operation as before, but this time, our error messages will include the exact position (line and column), where in a file an error occurred:

def parseSmilesLine = parseLine(smiles)

def bulkParseSmiles(ss: Seq[String]) =(ss traverse parseSmilesLine) ! 1

We use the ! operator to run the computation with an initial line index of 1.

### Parallelization and performance

At the beginning of this article we claimed that referentially transparent functions can safely be used in parallel algorithms. Indeed, we were able to parse a reasonably large file (about 10 MB) of SMILES strings with only a few lines of code using *Scala*’s parallel collections:

val path = "/home/ …"

def src = io.Source fromFile path

def ls = src.getLines.toSeq

val results: ValRes[Molecule] =(ls.par map parseSmiles seq).sequence

Function par transforms a collection to a parallel one, while seq transforms it back to a single threaded one. Calling map and sequence in succession does the same as calling traverse but the list is traversed twice. On the other hand, the implementation of traverse is too strict to be run in parallel; *scalaz* provides function parMap for that purpose, but this makes use of Futures which we will not describe here in more detail. Table 3 shows the time used to count the implicit hydrogen atoms in all molecules (about 350’000) in a sample SMILES file downloaded from the *ZINC* database [29] on a standard laptop with a quadcore processor, once when run in a single thread and once when run in parallel on all four cores. As can be seen, performance is increased by about a factor of two without any further tuning from our part.

**Table 3:** Performance of SMILES parsing

**Run**	**Singlethreaded**	**Multithreaded**	**Multithreaded**
			**(after profiling)**
1	17’149	10’295	7608
2	15’659	7123	5488
3	15’663	7248	5433
4	15’880	7508	5425
5	15’809	7669	5534
6	15’720	7197	5471
7	15’665	7174	5448
8	15’390	7296	5513
9	15’423	7687	5696
10	15’523	7564	5491
**average**	15’788	7676	5711

Time (in milliseconds) taken to parse part of the ZINC database containing about 350’000 structures on a quadcore laptop. The multi-threaded runs ran on all four cores without further optimization of *Scala*’s parallel collections settings.

We also used *VisualVM*[30] as a profiler to further improve the performance of our SMILES parser and our graph implementation. A couple of simple adjustments increased the performance by another 30%. Applying the *Flyweight* pattern to edges by caching the most commonly used ones as well as changing the order in the pattern match of the implicitHydrogens function each reduced the time needed to parse the *ZINC* library by about half a second. In the first case we could cache the commonly used edges’ hashCode which was otherwise reevaluated many times when they were being used as keys in the edge label map in our labeled graphs. In the second case we could restrict the sorting of a considerable amount of bond lists to only those cases where aromatic bonds were involved. Finally we saw that building the adjacency lists of our graphs took quite some time. This could be somewhat improved by changing their type from Array[Set[Int]] to Array[List[Int]]. It is not yet sure, whether we will have to undo this last change, since Sets have constant time random access, while for lists it is linear with respect to the list’s size. The effect on overall performance of our parser will not be very big if this change has to be undone in a future version.

Finally we tested the efficiency of parallelizing the parsing of large lists of SMILES strings on a hexacore processor supporting hyperthreading. The results are summarized in Table 4. The drop in efficiency and reduced speedup when running more than nine threads in parallel might be related to the computer’s hardware architecture or *Scala*’s implementation of parallel collections but we did no further investigations along those lines.

**Table 4:** Speedup and efficiency of parallelized SMILES parsing

**Number of Threads**	**Time [ms]**	**Speedup**	**Efficiency**
1	9493	1.00	1.00
2	5309	1.79	0.89
3	4134	2.30	0.77
4	2988	3.18	0.79
5	2785	3.41	0.68
6	2647	3.59	0.60
7	2273	4.18	0.60
8	2091	4.54	0.57
9	2037	4.66	0.52
10	2061	4.61	0.46
11	2061	4.61	0.42
12	2096	4.53	0.38

The test runs were performed on a hexacore processor supporting hyperthreading. Again our testing excerpt of the ZINC database was parsed using *Scala*’s parallel collections.

### Comparison with other open-source toolkits

In this section we are going to have a closer look at other cheminformatics toolkit implementations and compare their design with the one chosen for *chem*^*f*^.

#### Ouch: ouch uses chemical Haskell [12]

*Ouch* is the only other purely functional cheminformatics toolkit we are aware of. It is written in *Haskell* and is similar in functionality to *chem*^*f*^. It also provides SMILES parsing capabilities that result in a basic molecular graph. In addtion, *Ouch* supports output to several file formats as well as some nice algorithms for enumerating chemical structures which are described in more detail in the author’s blog [31]. There are several differences between *chem*^*f*^ and *Ouch* in terms of design decisions.

First, *chem*^*f*^ abstracts over the bond and atom types in its graph implementation through type parameters while in *Ouch* a molecule is simply a mapping from an integer to an atom plus a set of additional information in terms of molecular markers. All available information for a given atom is provided by the Atom data type which can again be annotated by various markers. This makes *Ouch*’s Atom and Molecule data types quite versatile but not really type safe. The presence or absence of a given type of marker is not visible at the type level, and algorithms that rely on certain markers will have to explicitly query the molecule and its atoms for their presence. Here we believe that the design of *chem*^*f*^ is somewhat superior in terms of flexibility and type safety. We can describe different aspects of a molecule simply by varying the type parameters of an LGraph and algorithms working on molecular graphs can define via the types of their parameters, which additional pieces of information about a molecule’s atoms and bonds are required. Parameterizing over the edge and node type of LGraph also allowed us to define some powerful type classes like Functor, Foldable, and Traverse for our labeled graphs. This makes many highly versatile higher order functions available for working with LGraphs as has been demonstrated when implementing a couple of basic operations on molecules.

Another difference is the implementation of the SMILES parser. *Ouch* makes use of the *parsec*[32] library of parser combinators, meaning that the low level parsing capabilities of *Ouch*’s parser did not have to be implemented from scratch. On the other hand, *Ouch* does not abstract over the return type of its parser. The parser simply reads a SMILES string and returns a Molecule. In our implementation we abstracted over the parser’s return type by means of the builder pattern. This leads to greater flexibility when accumulating the information available from a SMILES string and will make a possible future change in the representation of molecules easier. *Ouch*’s SMILES parser also handles the potential of failure during parsing differently: Errors and warnings are stored as markers together with the parsed molecule. While this also allows for the accumulation of error messages when several things go wrong, the presence or absence of errors is not visible at the type level and can only be determined upon inspecting the molecule’s set of markers. Our implementation describes the potential of failure already in the parser’s return type. This gives us once more access to many useful higher order functions provided by the Validation data type and its various type class implementations as we have shown when performing bulk operations. It also makes error handling in follow-up algorithms obsolete. This is demonstrated in the following code listing:

def formula (m: Molecule): Formula == m foldMap atomFormula

def parseFormula (s: String) =smiles(s) map formula

Here, function formula is only called if the parsing was successful. Its implementation does not have to concern itself with the possibility of failure during parsing, and it is therefore not necessary to query the molecule for the presence or absence of errors.

#### CDK: the chemistry developement kit [1]

*CDK* is an open-source cheminformatics and bioinformatics toolkit written in *Java*. It provides a plethora of chemistry-related data structures and algorithms as well as UI widgets that can be used for structure drawing and editing in graphical user interfaces. It also supports reading from and writing to many file formats typically used in chemical applications.

*CDK* was designed as a purely object-oriented toolkit, based on mutable data structures and subtyping for code reuse and loose coupling between components. The main data structures are defined as *Java* interfaces for which default implementations are provided. Algorithms typically operate on these interfaces which makes it possible to use them with tailor-made implementations. In *chem*^*f*^ we use type classes to achieve the same (or an even higher) degree of flexiblity. Theoretically, the algorithms provided by *CDK* could also be used from within *chem*^*f*^ by implementing the interfaces of the data structures in question. However, even *CDK*’s interfaces where designed with mutability in mind so that one has typically to provide getters (accessors) and setters (mutators) for all editable fields of a data structure. This of course does not go well with referential transparency and purely functional programming. It is also not always clear whether algorithms like substructure searching or ring detection mutate parts of the data structures they operate on. From a functional programmer’s point of view it might therefore be interesting to adjust *CDK*’s design so that two types of interfaces are defined for all data structures: One for accessing and one for mutating an object’s fields. Ideally, most algorithms would then work solely with the immutable interface of a data type, thereby guaranteeing via the type system that no inplace mutation will occur.

*CDK*’s SMILES parser is similar in functionality to our parser, however an algorithm for aromaticity perception is provided as well. *CDK*’s parser is also implemented via the *builder* pattern though its return type is always IMolecule. The builder is only needed as a factory to create a new instance of IMolecule when parsing a new string. It is also worth noting that *CDK*’s SMILES parser, written in an imperative style in *Java* consists of more than ten times the number of lines of code than our implementation. While fewer lines of code is not necessarly a sign of quality or efficiency, less boiler plate code can well increase both the readability and maintainability of the code base escpecially when the difference is a whole order of magnitude.

We planned to compare the efficiency of *CDK*’s SMILES parser with our implementation when parsing bulks of SMILES strings in parallel. However, we found that *CDK*’s parser is not referentially transparent and operates on mutable state internally. In order to parse SMILES strings in parallel we had to create a new instance of SmilesParser for every string to be parsed instead of using the same instance for all strings. Even then *CDK*’s SMILES parser failed with a NullPointerException at irregular occasions when we used the singleton instance of DefaultChemObjectBuilder as described in the parser’s documentation. The code we used in this test is listed below:

This demonstrates one of the major advantages of functional programming over classical object-oriented programming: Referentially transparent functions and immutable data structures can be used at will in parallel algorithms while with mutable data structures thread safety is a deliberate design decision and can be hard to implement and even harder to test.

## Conclusions

In this article, we described our first steps in putting together a purely functional cheminformatics toolkit in *Scala*. We showed how typical calculations required in chemical applications can be written with a drastically reduced amount of code due to the increase in abstraction and type-safety gained by applying typical concepts from functional programming. We also showed how the resulting referentially transparent functions and immutable data types can be used at no risk in all parts of client code including multi-threaded algorithms. We also compared our code with existing toolkits, and showed how it is superior in terms of type-safety and flexibility as well as – when compare to a typical object-oriented toolkit – thread safety. Last but not least, we experienced a drastic increase in productivity when programming in a purely functional way. Typically, code that compiled ran as expected and passed all unit tests at first take.

Of course our work is by far not finished here. In a next step we will focus on supporting other file formats in our toolkit before moving to file output and (SMILES) canonicalization. Especially the latter will be interesting to implement since it is known to be performance critical. Once canonicalization is implemented to our satisfaction we will move our focus towards data storage and experiment with our own implementation of a chemical database system that should also support substructure and similarity searches embedded in our computer aided drug design projects.

## Methods

### Benchmarking the SMILES parser

We used the scala.testing.Benchmark trait for benchmarking our application. For this task the block of code listed below was executed, either with or without using parallel collections.

The first run was typically slower by a factor of at least two than the following ones. This is a known effect due to *Java*’s Just In Time compilation [34].

## Competing interests

The authors declare that they have no competing interests.

## Authors’ contributions

SH did the programming and wrote the article. RR supervised the project. Both authors read and approved the final manuscript.

## Supplementary Material

Additional file 1 **chemf_src.zip.** This file contains the source code of the version of chem^f^ described in this article as an *SBT*[33] project. To try out some code samples, install *SBT* and start it from within the *chemf* directory. From within *SBT* start the console by typing console. Several functions are loaded and can be used out of the box: smiles(s) will parse SMILES string s, prettySmiles(s) will do the same but print the resulting molecule (or error messages) in human readable form. Click here for file

## References

[B1] Steinbeck C Han Y Kuhn S Horlacher O Luttmann E Willighagen E The Chemistry Development Kit (CDK): an open-source java library for Chemo- and bioinformatics J Chemical Inf Comput Sci 2003 43 2 493 500 10.1021/ci025584y PMC4901983 12653513

[B2] O’Boyle N Banck M James C Morley C Vandermeersch T Hutchison G Open babel: an open chemical toolbox J Cheminformatics 2011 3 33 10.1186/1758-2946-3-33 PMC3198950 21982300

[B3] The java programming language [http://www.java.com]

[B4] Bloch J Effective Java 2008 Boston: Addison-Wesley

[B5] Odersky M Future-proofing collections: from mutable to persistent to parallel Talk at Devoxx, Antwerp, Belgium 2010 [http://link.springer.com/chapter/10.1007âˆ–%2F978-3-642-19861-8_1]

[B6] Sondergaard H Sestoft P Referential transparency, definiteness and unfoldability Acta Informatica 1990 27 505 517

[B7] Odersky M Altherr P Cremet V Dragos I Dubochet G Emir B McDirmid S Micheloud S Mihaylov N Schinz M Stenman E Spoon L Zenger M An overview of the scala programming language Tech. rep., EPFL, Lausanne, Switzerland 2006

[B8] Peyton Jones S (Ed) Haskell 98 Language and Libraries – The Revised Report 2003 Cambridge, England: Cambridge University Press

[B9] Hughes J Why functional programming matters Comp J 1989 32 2 98 107

[B10] Höck S Riedl R Chimia 2012 66 3 132 134 22546259 10.2533/chimia.2012.132a

[B11] Wampler D Payne A Programming Scala 2009 Sebastopol: O’Reilly Media

[B12] Jankowski O Ouch uses chemical Haskell [https://github.com/odj/Ouch]

[B13] Weininger D Weinheim, Gasteiger J, Weinheim SMILES - a Language for molecules and reactions Handbook of Cheminformatics 2003 Germany: Wiley-VCH

[B14] Odersky M Spoon L Venners B Programming in Scala 2010 Walnut Creek, USA: Artima Press

[B15] FOLDOC: Free on-line dictionary of computing [http://foldoc.org/algebraic+data+type]

[B16] Moors A Piessens F Odersky M Generics of a higher kind Acm Sigplan Notices 2008 43 423 438

[B17] Odersky M Pimp my library 2006 [http://www.artima.com/weblogs/viewpost.jsp?thread=179766]

[B18] Oliveira BCdS Moors A Odersky M Type classes as objects and implicits Proceedings of the ACM International Conference on Object Oriented Programming Systems Languages and Applications 2010 Reno/Tahoe: ACM 341 360

[B19] Scalaz: Type classes and pure functional data structures for scala [http://code.google.com/p/scalaz/]

[B20] Lipovaca M Learn You a Haskell for Great Good 2011 San Francisco: No Starch Press, Inc.

[B21] OpenSMILES [http://www.opensmiles.org]

[B22] Barnard JM Weinheim, Gasteiger J, Weinheim Representation of molecular structures - overview Handbook of Cheminformatics 2003 Germany: Wiley-VCH

[B23] Cormen TH Stein C Rivest RL Leiserson CE Introduction to Algorithms 2001 Boston: McGraw-Hill Higher Education

[B24] Guha R Howard MT Hutchison GR Murray-Rust P Rzepa H Steinbeck C Wegner J Willighagen EL The blue obelisk - interoperability in chemical informatics J Chem Inf Model 2006 46 3 991 998 16711717 10.1021/ci050400b PMC4878861

[B25] Gamma E Helm R Johnson R Vlissides J Design Patters: Elements of Reusable Object-Oriented Software 1995 Boston: Addison-Wesley

[B26] McBride C Paterson R Applicative programming with effects J Funct Program 2008 18 1 13 [http://dx.doi.org/10.1017/S0956796807006326]

[B27] Gibbons J Oliveira Bcds The essence of the iterator pattern J Funct Program 2009 19 3-4 377 402 [http://dx.doi.org/10.1017/S0956796809007291]

[B28] Wadler P Monads for functional programming Advanced Functional Programming, First International Spring School on Advanced Functional Programming Techniques-Tutorial Text 1995 London: Springer-Verlag 24 52 [http://dl.acm.org/citation.cfm?id=647698.734146]

[B29] Zinc is not commercial [http://zinc.docking.org/]

[B30] VisualVM: All-in-one java troubleshooting tool [http://visualvm.java.net/]

[B31] Jankowski O Pharmash [http://www.pharmash.com/index.html]

[B32] Leijen D Meijer E Parsec: direct style monadic parser combinators for the real world Tech. rep. 2001

[B33] sbt, a build tool for Scala [https://github.com/harrah/xsbt/wiki/]

[B34] Goetz B Java theory and practice: Dynamic compilation and performance measurement [http://www.ibm.com/developerworks/library/j-jtp12214/]

