# Supplementary material for: chemf: A purely functional chemistry toolkit
Source: J Cheminform. 2012 Dec 20;4:38. doi: 10.1186/1758-2946-4-38 (PMC3660204; doi:10.1186/1758-2946-4-38)
Supplement: Additional file 1 — chemf_src.zip. This file contains the source code of the version of chemf described in this article as an SBT[33] project. To try out some code samples, install SBT and start it from within the chemf directory. From within SBT start the console by typing console. Several functions are loaded and can be used out of the box: smiles(s) will parse SMILES string s, prettySmiles(s) will do the same but print the resulting molecule (or error messages) in human readable form. [file 1758-2946-4-38-S1.zip › chemf/src/main/scala/chemf/parser/fchem_publi.pdf]

(1)Life Sciences Department, Kings College London, Cornwall House,Waterloo Road, London, UK

(2)Department of Zoology, Cambridge, Waterloo Road, London, UK

(3)Marine Ecology Department, Institute of Marine Sciences Kiel, Düsternbrooker Weg 20, 24105 Kiel, Germany

*Background: Text for this section of the abstract.

*Results: Text for this section of the abstract …

*Conclusions: Text for this section of the abstract …

*Background

[?, ?, ?, ?, ?, ?, ?, ?, ?, ?, ?, ?, ?, ?]

*Results and Discussion

*A purely functional SMILES parser We'd like to show some of the aspects and benefits of functional programming by implementing a basic SMILES-parser, that accumulates molecular data while parsing a single one-line string. This is done without using mutable state whatsoever. While at first this might seem limiting, we will see later, that such a parser can be more flexible and composable than a comparative imperative implementation.

*First things first: The main parser layout Parsing a SMILES strings means interpreting a single line of data one character at a time, therewhile accumulating an increasingly complex molecular structure. We therefore need two things: A data type representing the accumulated molecule and a finite state machine (FSM) that on one hand keeps track of the accumulated molecule and on the other hand provides a method 'next' that takes a single character as its argument and returns a new FSM depending on prior characters and the so far accumulated molecular state. One minor detail about accumulating the molecular state: Instead of keeping track of an actual molecular graph consisting of atoms and bonds, we rather accumulate a state function, that takes a molecule as its input and returns a new molecule as its output. That way, several strings can be parsed and the resulting state functions combined at will giving us maximum flexibility. In order to get a 'real' molecule, we just have to give the empty molecule as a parameter to the state function and get the parsed molecule as our result.

```
object FSMParser {

  def parseState[S] (s: String, init: FSM[S]): S =
    s.foldLeft(init)(_ next _).state

  /**
   * Finite state machine
   */
  sealed trait FSM[+S] {
```

```
    /**
     * The next FSM, depending on the new input
     */
    def next (c: Char): FSM[S]

    /**
     * The state accumulated so far
     */
    def state: S
  }

  /**
   * FSM factory method. Value s is given as lazy parameter
   * since it is not in all cases necessary to actually evaluate it.
   */
  def fsm[S] (n: Char  FSM[S], s:  S): FSM[S] = new FSM[S] {
    def next(c: Char) = n(c)
    def state = s
  }

}
```

Parsing a string of characters is now as simple as writing

```
def parseState (s: String): StateTrans =
  s.foldLeft (initial)(_ next _).stateTrans
```

The foldLeft function iterates over a given sequence (a String in Scala can be handled like a sequence of characters) taking an initial value and an accumulating function as its argument. It is one of two (the other being foldRight) general-purpose functions for collapsing collections down to a single value.

If we want to directly get a molecule as our result we can define

```
def parse (s: String) = parseState(s)(Mol.empty)
```

If on the other hand we'd like to parse two separate strings and combine them in a single state transformer we could do the following:

```
def parseTwo (a: String, b: String) = parseState(a) |+| parseState(b)
```

The |+| operator is defined in scalaz and used two values to give a new value of the same type. For this to work we need to provide a Monoid type class in the implicit scope:

```
implicit val StateTransMonoid = new Monoid[StateTrans] {
  val zero: StateTrans = identity
  def append (a: StateTrans, b: => StateTrans) = a andThen b
}
```

The identity function just returns its single argument, while the andThen function combines to functions by using the result of the first as the input parameter of the second.

Note how flexible we are at handling the results of our parsings. We can parse many strings and combine the resulting state transformers without ever having to work with an implementation of an actual molecular graph. So far we have just combined functions without caring about chemistry at all. No matter how we are going to change the implementation of Mol or the concrete type of StateTrans (we are going to adjust both several times), we will never ever have to change our parsing functions as long as we can provide a Monoid for type StateTrans and define an initial value for our FSM.

As a side not, we could even add one more layer of abstraction by adding a type parameter to FSM and StateTrans resulting in StateTrans[A] = A => A. Thus we would decouple the whole parsing issue completely from molecules and chemistry whatsoever. In a concrete SMILES parser implementation one would then have to limit the possible type parameters by requiring an AsMolecule type class as an implicit parameter that provides the functions necessary to actually accumulate a molecule. Thus we would gain complete liberty about our molecular graph implementation. This approach is somewhat similar to implementing a common interface in a language like Java using the mechanisms of subtyping and indeed we could use this approach in Scala as well. Using type classes is a somewhat more functional approach, which can from our experience result in greater flexibility than using a rigid class hierarchy.

*A first implementation: Parsing a single chain of atoms The following listing shows the FSM implementation of a very simple parser that only reads single letter atoms and connects them via single bonds. This parser only has three different states: The initial state, an invalid state (right now, this is no real state at all but just an exception being thrown; we will change this later on) and a state that reads a single character and either returns a new single character reading state or the invalid state if the character in question is not found.

```
def invalid: FSM = throw new Exception("Invalid smiles string")

val initial = fsm (char (zeroTrans), zeroTrans)

def char (last: StateTrans)(c: Char): FSM = {
  def nextChar (s: StateTrans) = fsm (char(s), s)

  uniqueChars get c fold (s => nextChar(last |+| s), invalid)
}

private val uniqueChars: Map[Char, StateTrans] = Map (
  'C' -> addAtom (Atom fromElement C),
  'B' -> addAtom (Atom fromElement B),
  'O' -> addAtom (Atom fromElement O),
  'N' -> addAtom (Atom fromElement N),
  'I' -> addAtom (Atom fromElement I),
  'S' -> addAtom (Atom fromElement S),
  'F' -> addAtom (Atom fromElement F)
)
```

We use a Map from characters to StateTrans here to simplify our code. The method char looks up the character in question in the map. The lookup function 'get' returns an optional value of type Option[StateTrans]. This is an algebraic data type with two possible values: In case of a successful lookup, the return value is of type Some(s), s being the found StateTrans. In case of a failure the returned value is None (a constant). This is the functional way of handling a simple case of failure in a calculation. Instead of returning null, which is what a HashMap in Java would do when a key is not found upon lookup, the return type of the get function forces the programmer to deal with the possibility of failure. Not doing so would result in a compilation error. The type system guarantees that the get function *always* returns a value of the given type, without requiring the programmer to think about possible NullPointerExceptions. In order to get a value out of an Option, we can use the fold function, a higher-order function that takes two paramters: A function of arity one, which is called if the Option has an actual value stored, or an alternative value that is returned in case of a None. The fold function is not part of the Scala standard library but is provided by the scalaz library.

In our case, we just generate an new FSM from the last TransState if the lookup was successful, and we return the invalid FSM, if the lookup failed, in which case the SMILES string contained a character our parser could not handle.

For the accumulated molecular state, we keep things very basic here. Since our molecule consists only of a single chain of atoms connected by single bonds, we can use a simple List of atoms to represent our molecule:

```
type Mol = List[Atom]
def addAtom (a: Atom): StateTrans = a :: _
val empty: Mol = Nil
```

Instead of defining a new class for our molecule, we just use a type alias. Method addAtom just prepends the latest atom to the list (a constant time operation). The empty molecule is represented by the empty list Nil.

*Supporting different bond types In this section we are going to enhance our SMILES parser, so that it can also handle double, triple, and aromatic bonds. For this we define an algebraic data type BondType that consists only of the four bond types as constants:

```
sealed trait BondType
```

```
object BondType {
  case object Single extends BondType
  case object Double extends BondType
  case object Triple extends BondType
  case object Aromatic extends BondType
}
```

We also define a type alias for a chemical bond which is just a pair of an Edge in a graph (usually consisting of two integer indices denoting the connected nodes) and a BondType:

```
type Bond = (Edge, BondType)
```

In our FSM we only have to add the following for entries to the uniqueChars map:

```
private val uniques: Map[Char, StateTrans] = Map (
  ...
  '-' -> setBond (Single),
  '=' -> setBond (Double),
  '#' -> setBond (Triple),
  ':' -> setBond (Aromatic)
)
```

Our implementation of the molecular state gets a bit more complex. It is shown in the listing below:

```
case class Mol (
  count: Int,
  bonds: List[Bond],
  atoms: List[Atom],
  bond: Option[BondType]
) {
  def setBond (b: BondType):Mol = bond match {
    case Some(_) => throw new Error("Bond set twice")
    case None => copy(bond = Some(b))
  }

  def addAtom (atom: Atom): Mol =
    Mol(count + 1, bondTo (count, count - 1), atom :: atoms, None)

  def bondTo (from: Int, to: Int): List[Bond] = {
    def bnd = (Edge(from, to), bond getOrElse BondType.Single)
    atoms.headOption fold (_ => bnd :: bonds, bonds)
  }
}
```

We now keep track of the number of atoms in the molecule, as well of all atoms and bonds, both implemented as Lists. In addition we can set an optional BondType. If a new atom is added, and the BondType was set, we use this BondType to connect the new atom with the last on the stack. If no BondType was set, we connect them via a single bond.

*Results sub-heading *This is a sub-sub-heading Sub-sub-sub-headings are made with the

subsubsection command. pb at end of lines ensures correct paragraph spacing.Text for this sub-sub-section ...*Another sub-sub-sub-heading Text for this sub-sub-section ...

*Another results sub-heading Text for this sub-section ...

*Yet another results sub-heading Text for this sub-section. More results ...

*Conclusions Text for this section ...

*Methods *Methods sub-heading for this section Text for this sub-section ...

*Another methods sub-heading for this section Text for this sub-section ...

*Yet another sub-heading for this section Text for this sub-section ...

*Authors contributions Text for this section ...

*Acknowledgements Text for this section ...

*Figures *Figure 1 - Sample figure title A short description of the figure content should go here.

*Figure 2 - Sample figure title Figure legend text.

*Tables *Table 1 - Sample table title Here is an example of a *small* table in LaTeX using `\tabular{...}`. This is where the description of the table should go.

My Table		
A1	B2	C3
A2	...	..
A3	..	.

*Table 2 - Sample table title Large tables are attached as separate files but should still be described here.

*Additional Files *Additional file 1 — Sample additional file title Additional file descriptions text (including details of how to view the file, if it is in a non-standard format or the file extension). This might refer to a multi-page table or a figure.

*Additional file 2 — Sample additional file title Additional file descriptions text.
